# Potential therapeutic effects of milk-derived exosomes on intestinal diseases

**DOI:** 10.1186/s12951-023-02176-8

**Published:** 2023-12-20

**Authors:** Zhifu Cui, Felix Kwame Amevor, Xingtao Zhao, Chunyan Mou, Jiaman Pang, Xie Peng, Anfang Liu, Xi Lan, Lingbin Liu

**Affiliations:** 1 College of Animal Science and Technology, Southwest University, Chongqing, P. R. China; 2 Farm Animal Genetic Resources Exploration and Innovation Key Laboratory of Sichuan Province, Sichuan Agricultural University, Sichuan, P. R. China; 3 College of Pharmacy, Chengdu University of Traditional Chinese Medicine, Sichuan, P. R. China; 4 College of Animal Science and Technology, Chongqing Key Laboratory of Forage & Herbivore, Chongqing Engineering Research Center for Herbivores Resource Protection and Utilization, Southwest University, Beibei, Chongqing, 400715 P. R. China

**Keywords:** Milk-derived exosome, Intestinal Disease, Inflammation, Drug delivery, Nanocarrier

## Abstract

Exosomes are extracellular vesicles with the diameter of 30 ~ 150 nm, and are widely involved in intercellular communication, disease diagnosis and drug delivery carriers for targeted disease therapy. Therapeutic application of exosomes as drug carriers is limited due to the lack of sources and methods for obtaining adequate exosomes. Milk contains abundant exosomes, several studies have shown that milk-derived exosomes play crucial roles in preventing and treating intestinal diseases. In this review, we summarized the biogenesis, secretion and structure, current novel methods used for the extraction and identification of exosomes, as well as discussed the role of milk-derived exosomes in treating intestinal diseases, such as inflammatory bowel disease, necrotizing enterocolitis, colorectal cancer, and intestinal ischemia and reperfusion injury by regulating intestinal immune homeostasis, restoring gut microbiota composition and improving intestinal structure and integrity, alleviating conditions such as oxidative stress, cell apoptosis and inflammation, and reducing mitochondrial reactive oxygen species (ROS) and lysosome accumulation in both humans and animals. In addition, we discussed future prospects for the standardization of milk exosome production platform to obtain higher concentration and purity, and complete exosomes derived from milk. Several in vivo clinical studies are needed to establish milk-derived exosomes as an effective and efficient drug delivery system, and promote its application in the treatment of various diseases in both humans and animals.

## Introduction

Intestinal diseases such as inflammatory bowel diseases (IBD), necrotizing enterocolitis (NEC), colorectal cancer (CRC), intestinal ischemia and reperfusion injury (IR) are generally characterized by clinical symptoms, including intestinal dysfunction and injury, intestinal inflammation, intestinal mucosal immune disorder, and microbiome imbalance [1–4]. Exosomes are cell-derived vesicles which are widely involved in the progression of intestinal diseases as well as play an important role in disease diagnosis and also serve as drug carriers [1–4]. Exosome is obtained via merging multivesicular bodies and is the latest family member of bioactive vesicles that play functional roles in promoting cell-cell communication [5]. In addition, exosomes were reported to be originated from endosome, in consequence contained many biomolecular elements based on their cell of origin, hence, they are described as a ‘‘fingerprint’’ of the origin of the cell [6].

### Biogenesis, secretion and structure of exosomes

**Fig. 1:**
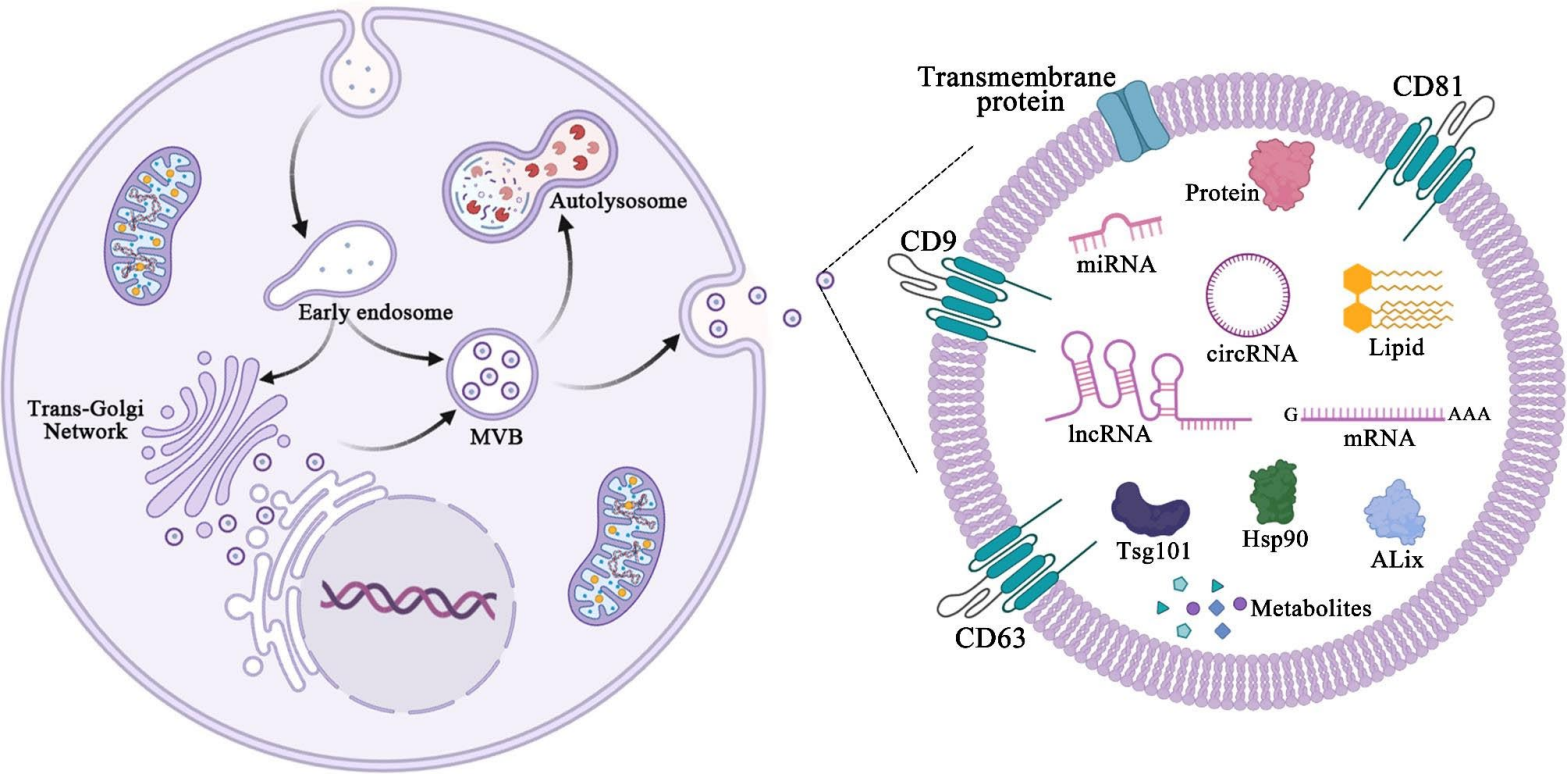
Exosomes biogenesis, secretion and structure

Attention on exosome research has broaden due to their description in antigen-presenting cells as well as the reports that they play active role in enhancing immune response in vivo [7]. Exosomes are membranous vesicles with a diameter ranging from 30 ~ 150 nm. They are released outside the cell after the cellular polyvesicles fused with the cell membrane originating from the endocytic pathway through the inward budding of the plasma membrane [8, 9]. This process generates the early endosome, which by a subsequent inward budding process creates the multivesicular bodies (MVB) characterized by the presence of vesicles in their lumen (intraluminal vesicles, ILV). The MVB is responsible for releasing exosomes by the transport and fusion of MVB with the plasma membrane, thus, the ILV are released into the extracellular space and then referred to as “exosomes” [10–12]. The exosomes are coated with bilayer phospholipid membranes and contain high levels of cholesterol, sphingomyelin, ceramides, and short/long chain saturated fatty acids [10, 13]. In addition, the exosomes contain cell-specific proteins, lipids, and nucleic acids (nucleic acids, namely mRNA, noncoding RNA species, and DNA) [14, 15]. The exosome biogenesis is the mechanism for protein quality control. Once the exosomes are released, they are involved in several activities such as extracellular matrix remodeling, as well as serving as signaling molecule to other target cells, thereby altering their functions [16, 17], however, their effects on target cells vary due to the differences in the expression profile of receptors on the cell surface. Such functional heterogeneity cause exosomes to modulate cell survival induction, apoptosis, and immune regulation in different target cell types [11]. In addition, exosome heterogeneity increases the functional diversity and complexity of exosomes in an intercellular communication. Exosomes originating from different cell types may have different compositions, however, they possesses similar conserved proteins such as CD63, CD81, CD9, etc. [18] (Fig. 1). In general, exosomes are found in a variety of living cells including dendritic cells (DCs), lymphocytes, epithelial cells, endothelial cells, etc. They are also found in the body fluids of eukaryotes, such as blood, urine, saliva, cerebrospinal fluid and emulsion [19, 20]. Studies have reported that exosomes are also involved in progress of diseases, such as neurodegenerative diseases [21–23], obesity and diabetes [24–27], cancer [28–30], etc., as well as play important role in disease diagnosis and also serve as a drug carrier [31–36].

### Current methods for the extraction of milk-derived exosomes

**Fig. 2:**
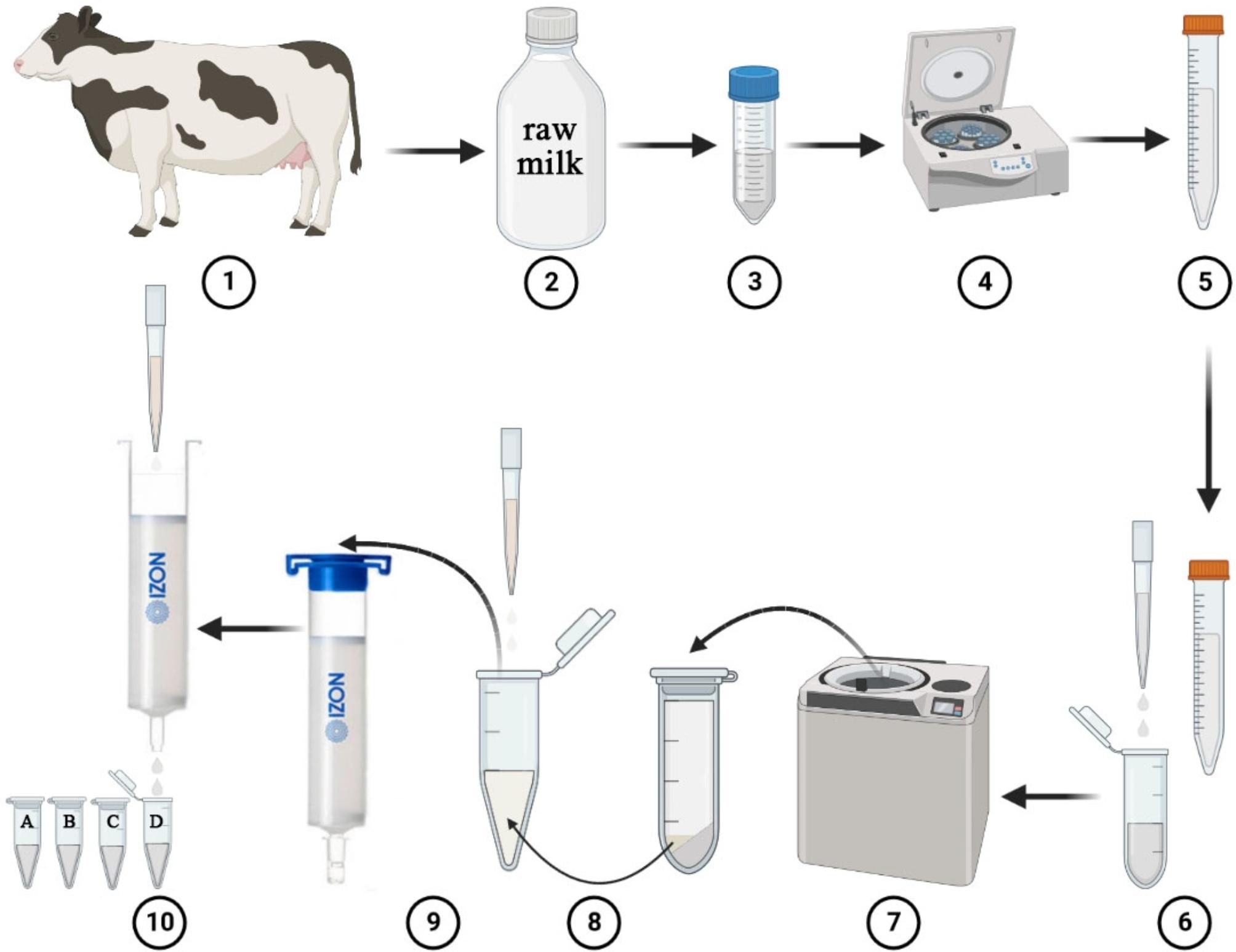
The isolation process of bovine milk-derived exosome. ①: Dairy cow; ②: Raw milk; ③: Divide milk into EP tubes; ④: Centrifuge at 12,000×g, 4 ℃ for 30 min to remove the remaining fat and cell debris; ⑤: Skimmed milk; ⑥: Skimmed milk was transferred into an ultracentrifugation tubes; ⑦: Centrifuge at 150,000×g, 4 ℃ for 2 h; ⑧: The exosome pellet was collected and transferred into a low binding tube and resuspended in phosphate buffer solution (PBS) to 500 µL; ⑨: the suspension sample was loaded on a qEV original 35 nm SEC column; ⑩ After 3 mL of void volume, 4 fractions (A, B, C, D) of each 500 µL were immediately collected

Milk obtained from animals or humans is a complex, heterogeneous fluid containing a non-nutritive, bioactive extracellular vesicle known as exosome. Milk-derived exosomes (MDEs) are very difficult to characterize because of the lack of effective and efficient standardized methods used for milk pre-processing, storage, and exosome segregation [37]. Several techniques such as ultracentrifugation, size exclusion chromatography (SEC), and density gradient centrifugation (DGC) are currently available for separating exosomes from milk [38, 39]. Among these exosome isolation methods, ultracentrifugation (differential centrifugation) is the most standard, and with this procedure, raw milk can be centrifuged at approximately 2,000×g to remove fat globules, dead cells, and bulky apoptotic debris. Thereafter the exosomes are precipitated approximately at the speed of 100,000 to 150,000×g [37, 40–42]. Then the exosome pellets are loaded on a SEC column to get four fractions of exosomes for further characterization and analysis [42]. SEC is the method used to extract milk exosomes according to the size of the exosomes. In present studies, the extraction of milk exosomes by the SEC method is mostly combined with the ultracentrifugation method [42, 43]. Studies have extracted milk exosomes by using ultracentrifugation method combined with the SEC and DGC methods [44, 45]. In our previous study, we successfully separated bovine milk-derived exosomes using the ultracentrifugation method combined with SEC method (Fig. 2). At present, isolating exosomes from milk using these methods may be superior compared to the single method. For the DGC method, samples are added into an inert gradient medium for centrifugal sedimentation [46]. Various ingredients of the sample will settle on their respective isodensity zone under a centrifugal force, after which the exosomes will be separated from each other. In addition, the sucrose gradient centrifugation could effectively avoid the co-precipitation of nucleosomal fragments, apoptotic bodies, or protein aggregates [47] to achieve greater separation efficiency than the conventional method, thereby providing exosomes with high purity [48].

Furthermore, some receptor molecules such as CD9/63/81 on the membrane surface can be utilized to isolate exosomes by employing immuno-affinity capture method [49, 50]. The most commonly used immunocapture kits are enzyme-linked immunosorbent assay (ELISA), and in recent years, immunomagnetic beads are also becoming popular [51]. Microfluidic technology is a recently developed technique which is specifically used for high demanding separation tasks. At present, microfluidic technology is mainly divided into three categories: these are separation based on size, separation based on immunoaffinity, and dynamic separation [52]. To isolate exosomes from various milk fractions, other studies have introduced a novel approach based on natural nanosolid cellulose nanofibers (CNFs) and short time low gravity centrifugation, as well as encasing exosomes with flexible and entangled network of CNFs forms nanoporous [53].

### Methods and markers for the identification of exosomes

**Fig. 3:**
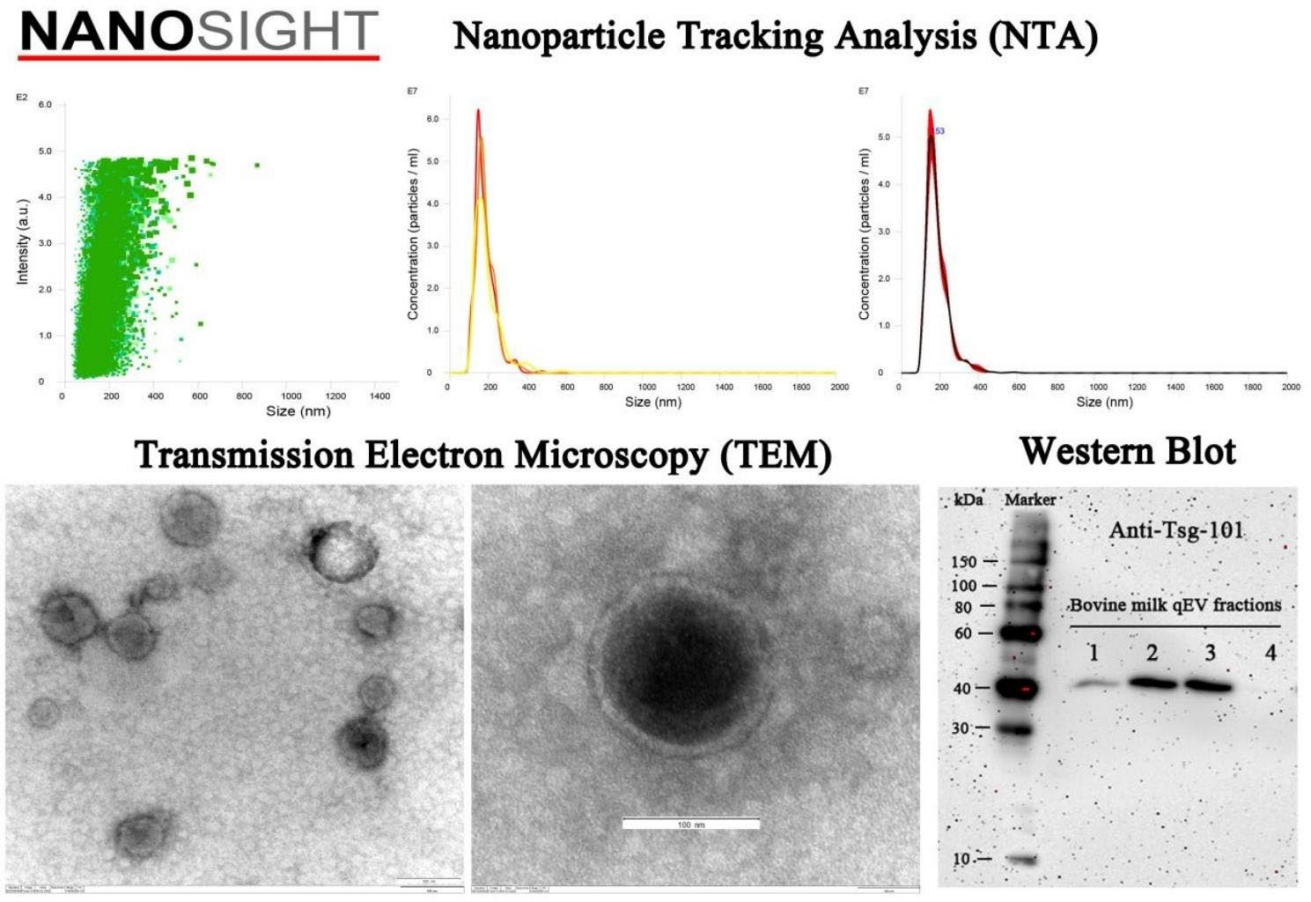
Bovine milk-derived exosomes were characterized using NTA, TEM, and WB (Zhifu Cui unpublished data)

Generally, exosomes are characterized using nanoparticle tracking analysis (NTA) [54–58], transmission electron microscopy (TEM) [54–58], western blot (WB) [54, 55, 57, 58], flow cytometry (FCM) [56–58], and PKH67 fluorescent labeling [59, 60]. The size distribution and concentration of particles in exosomes are analyzed by NanoSight instrument. The fractions are diluted to 1:25 − 1:1000 fold with PBS to keep the number of particles in the field between 50 and 200/frame. For TEM analysis, a total of 50 µL purified exosome are pipetted on Parafilm® and immediately adsorbed to an Athene old 400 mesh copper grid coated with 1% Piolofom® in chloroform (w/v), and then incubated for 5 min at RT. The grid will then be carefully washed twice with distilled water and negatively stained with 50 µL of 2% uranyl acetate (w/v). Then the samples can be viewed using the Zeiss EM 10^9^ TEM. The WB can also be performed to verify exosomal markers, such as CD9/63/81 [61–65], tumor susceptibility gene 101 (Tsg101) [63, 66–69], heat shock 70 kDa protein (HSP70) [70–72], Alix [73–75], and Flotillin 1 [74, 76, 77]. FCM is mostly used for exosome characterization. In brief, the freshly isolated exosomes are diluted in the 0.22 μm-filtered PBS and then stained under sterile dark conditions with green-RNA-binding, a liposoluble fluorophore SYTO that can penetrate the exosomal membrane. Before the samples can be loaded into the flow cytometer CytoFlex S, they are vortexed and bathed at 37 ℃ in the dark for 30 min, and then can be visualized using the CytExpert software [78]. PKH67 is a novel dye that can fluorescently label exosomes by binding to lipid molecules in the exosomal membrane structure [60]. Several studies on cellular uptake have co-cultivated cells with PKH67-labeled exosomes [79–81], and the results showed that PKH67-labelled breast milk exosomes can be taken up by macrophages [82] and IECs [80]. In our previous study, bovine milk-derived exosome was characterized by methods such as NTA, TEM, and WB (Fig. 3).

## Therapeutic effects of milk-derived exosomes on intestinal Diseases

The intestinal tract plays major role in nutrient digestion, absorption, as well as serves as an immune and endocrine organ. The intestinal tract is the main immune defense barrier which is composed of the mucosal immune system and intestinal epithelial cells (IECs). Exosomes has key physiological and pathological implications on gut-related diseases, such as Inflammatory Bowel Disease (IBD) [83–86], colitis [87, 88], Colorectal Cancer (CRC) [89–93], Necrotizing enterocolitis (NEC) [94–97], and intestinal ischemia and reperfusion (IR) injury [98–100]. Mammalian milk is rich in exosomes, which play key roles in intestinal development, prevention of excessive inflammation, maintenance of intestinal epithelium integrity, and can also be used for disease treatment [101–105]. Mammalian breast milk exosomes transport proteins and nucleic acids to the neonatal intestinal system, thereby protecting them from acidity degradation and digestion, and also promote their intestinal structural integrity and absorption, hence, milk-derived exosome promotes intestinal development [105]. A study analyzed the expression of miRNAs in the human breast milk and reported high expression levels of immune-related miRNAs in the first 6 months of lactation, which regulated the development of intestinal immune system in the infants [106]. Other studies have shown that milk derived exosomes are rich in transforming growth factor β (TGF-β), which perform significant role in the development of intestinal barrier function, the production of immunoglobulin A (IgA) and mucosal immunity during the infancy period [107]. Reports have indicated that breast milk-derived exosomal circRNAs bind to related miRNAs promote IEC proliferation and migration through the vascular endothelial-derived growth factor (VEGF) signaling pathway, thereby promoting the development of intestinal tract [108].

In addition, studies have revealed that porcine milk-derived exosomes promote proliferation and intestinal development of porcine’s small intestinal cells, improve the expressions of caudal-related homeobox transcription factor 2 (CDX2), proliferate cell nuclear antigen (PCNA) and type 1 insulin-like growth factor receptor (IGF-1R) genes in porcine small intestine cells, as well as inhibit the expression of tumor suppressor gene p53 [109]. CDX2 is a gut specific transcription factor that is directly involved in the intestinal development and maintenance of intestinal phenotypes [110]. It was reported that porcine milk-derived exosomal circ-XPO4 plays a crucial role in the intestinal acquired immunity and mucosal homeostasis via inhibiting the expression of miR-221-5p, promoting the expression of polymeric immunoglobulin receptors and the production of intestinal IgA [111]. Porcine milk-derived exosomal miRNAs were found to alleviate deoxynivalenol (DON) induced intestinal mucosal damage in mice by promoting cell proliferation and inhibiting apoptosis [112]. Specifically, porcine milk-derived exosomal miR-4334, miR-219 and miR-338 attenuate lipopolysaccharide (LPS)-induced intestinal cell inflammation and apoptosis, and relieve intestinal damage, as well as maintain the intestinal epithelial integrity via inhibiting the activation of Toll-like receptor 4 (TLR4)/ NF-kappaB (NF-κB) and p53 signaling pathways [113].

Several studies have shown that bovine milk-derived exosomes escape the absorption in the digestive tract, and induce changes in the intestinal microbial community, leading to the enrichment of the polymorphisms and mutations of the rectal bacteria in mice [114], improve the atrophy of the intestinal villus in mice [115], and also increase the production of the intestinal mucus and enhanced tight junction protein expression via miRNAs and TGF-β to aid in the restoration of the intestinal barrier function induced by diseases [104]. Bovine milk-derived exosomes change the intestinal microbial community of mice and promoted the communication between the host and bacteria [114]. It was also reported that bovine milk-derived exosomal miRNAs are involved in immune response, growth and development, which is beneficial to dairy cows and the maturation of the intestinal structure of the neonate [116]. Another study have indicated that the oral administration of bovine milk-derived exosomes cause senescence of the primary intestinal tumors and accelerate cancer metastasis in mice [117], in addition, yak milk-derived exosomes were reported to promote proliferation and survival of IECs under hypoxic environment [72]. Goat milk-derived exosomes can be used as a natural probe to detect inflammatory process. Injection of goat milk-derived exosomes in peritonitis mice significantly increased the exosomal content of the intestine [118]. Rat milk-derived exosomes also significantly increased the expression of PCNA and leucine-rich repeat-containing G-protein coupled receptor 5 (Lgr5) genes, as well as enhance the activity of IECs [119]. Other studies have shown that giant panda milk-derived exosomes promote the development of the intestinal immune system and absorption in newborn cubs [120]. Exosomes play significant physiological and pathological role on proper functioning of the intestine. The exosomes affect the progression of intestinal inflammatory response following the beginning of related pathologies. The existence of many uptake exosomal mechanisms of the intestine promotes the alleviation of pathological conditions of the intestine [102, 121]. Cells communicate with each other by releasing exosomes that transfer their composition, such as nucleic acids, proteins, and lipids, to the nearby cells, hence play important function in several pathophysiological processes [122, 123]. For instance, during pathogenic bacteria infection, exosomes are secreted by infected cells to affect the innate immune responses of the neighbouring cells to the infection. These vesicles can release different biological fluids to allow changes in the content of the exosome to help in the discovery of non-invasive biomarkers related to inflammatory bowel disease and infectious diseases [122, 123]. Studies also indicated that exosomes could be utilized as a vaccine for boosting the immune system to get rid of various pathogenic bacteria and to attenuate intestinal damage [122, 123].

### Inflammatory bowel Disease

Inflammatory Bowel Disease (IBD) is a recurrent and lifelong disease that includes Ulcerative colitis (UC) and Crohn’s disease (CD) characterized by chronic, recurrent, and nonspecific intestinal inflammation [124–126]. The clinical manifestations of IBD are persistent or recurrent abdominal pain, diarrhea, fever, rectal bleeding and other symptoms. The diagnosis and treatment of IBD are complicated [127, 128]. Currently, the pathogenesis of IBD is related to several factors such as genetics, intestinal mucosal barrier damage, intestinal inflammation, gut dysbiosis and intestinal mucosal immune disorder [129–132]. Several studies have shown that milk-derived exosomes play crucial roles in the prevention and treatment of IBD by participating in the interaction and communication of IECs-immune cell-intestinal flora to regulate the immune response and intestinal homeostasis, as well as attenuate intestinal inflammation [133–135].

Studies have also shown that bovine milk-derived exosomes alleviate UC by reducing inflammatory response through inhibition the production of pro-inflammatory factors via TLR4-NF-κB signaling pathway and the activation of nod-like receptor family pyrin domain containing 3 (NLRP3) inflammatories, attenuating cytokine production disorder and restoring the balance between the interleukin-10^+^Foxp3^+^ regulatory T (Treg) cells and T helper type 17 (Th17) cells in the inflamed colon, and also restoring the α-diversity of gut microbiota effectively, as well as regulating intestinal immune homeostasis [136]. Bovine milk-derived exosomes were also reported to alleviate dextran sodium sulfate (DSS)-induced IBD in mice by restoring the intestinal impermeability and promoting mucin secretion by regulating the intestinal microbial flora, reducing inflammation by down-regulating the expression of colitis related miR-125b, increasing the expression of anti-inflammatory protein such as TNF-alpha-induced protein 3 (TNFAIP3, A20), reducing the production and release of pro-inflammatory cytokines and increasing the production of anti-inflammatory cytokines to restore the structure and integrity of the colon [137]. A study investigated the therapeutic effect of cow and human milk derived exosomes on colitis mice, and they have found that the oral administration of cow and human milk-derived exosomes play an anti-inflammatory and therapeutic role to reduce the severity of DSS-induced UC in mice by down-regulating the expression of pro-inflammatory cytokines tumour necrosis factor alpha (TNF-α) and interleukin 6 (IL-6), and also up-regulates the expression of TGF-β [138]. Oral administration of bovine milk-derived exosomes alleviates clinical symptoms and colon damage in mice with UC induced by DSS by attenuating oxidative stress, as well as reducing the expression of inflammatory cytokines and chemokines in the colon [139]. In addition, bovine milk-derived exosomes can attenuate DSS-induced UC in the mice by remodeling and optimizing the abundance of intestinal flora, regulating intestinal gene expression, and restoring the structure and integrity of the intestinal surface epithelium [140]. Moreover, bovine milk-derived exosome was reported to help in the restoration of metabolic abnormalities induced by DSS-induced UC in the mice, and also prevent intestinal inflammation by regulating lipid and amino acid metabolism, thereby providing new insights into the identification and utilization of lactation-derived exosomes as potential regulators for the prevention and treatment of IBD [141]. Goat milk-derived exosomes were also reported to show anti-inflammatory and immunomodulatory effects, hence can reduce LPS-induced inflammation of the porcine jejunal epithelial cells (IPEC-J2 cells) and also restore cellular homeostasis by decreasing the level of expressions of IL18, IL12p40, matrix metalloproteinase 9 (MMP9) and nitric oxide synthase (NOS2), but increase the level of expressions of mucin 2 (MUC2), epstein-barr virus-induced gene 3 (EBI3), and IL-8 [142].

### Necrotizing enterocolitis (NEC)

Necrotizing enterocolitis (NEC) is one of the most devastating diseases of premature infants, characterized by high morbidity and mortality rates [143, 144]. Therefore, it is urgent to develop effective treatments for this devastating condition. Breast milk, which has been known for decades to have health benefits, contains large amounts of exosomes and has the potential to treat NEC diseases [145]. Breast milk has been shown to reduce the incidence of NEC, however, NEC condition is rare in infants whose diets contain breast milk [146]. Compared with formula milk, breast milk feeding reduces the risk of NEC [147]. Various studies have shown that the activation of TLR4 induced-inflammation inhibits IEC proliferation, reduces intestinal microcirculation, and promotes the occurrence and progression of NEC [148, 149], however, other studies have reported that epidermal growth factor in breast milk inhibits TLR4 signaling, protects IECs from apoptosis, promote intestinal cell proliferation, and inhibit the occurrence of NEC [150]. Breast milk-derived exosomes have been shown to prevent NEC in premature infants [151]. In vitro and in vivo studies have demonstrated that peptides highly enriched in milk-derived exosomes can reduce ileal damage by promoting the intestinal cell proliferation and migration, which may be an effective preventive method for NEC [152]. Human breast milk-derived exosomes were found to protect the intestinal stem cells from oxidative stress damage via the Wnt/β-catenin signaling pathway to prevent and treat the development of NEC [153]. Reports indicated that the incidence of NEC is 0% in breastfed pups, and human breast milk-derived exosomes significantly increased the IEC proliferation and also inhibited apoptosis, as well as reduced the incidence and severity of NEC [154]. Other studies have reported that human breast milk-derived exosomes exert protective effect on IECs, and also promote cell viability by alleviating oxidative stress, thereby preventing the occurrence of NEC and intestinal injury [155]. Human milk-derived exosomal lncRNAs and mRNAs prevent the occurrence of NEC by promoting intestinal tissue proliferation and development, reducing intestinal tissue necrosis and epithelial injury, as well as reducing the severity of NEC through the JAK-STAT and adenosine monophosphate-activated protein kinase (AMPK) signaling pathways [95]. Human milk-derived exosomal lipids reduce the severity of NEC through the extracellular signal-regulated protein kinase/mitogen activated protein kinase (ERK/MAPK) pathway to rescue the apoptosis and migration inhibition of IECs induced by LPS [156]. In other studies, human milk-derived exosomes were reported to alleviate hypoxia and LPS-induced NEC inflammation, mucosal damage, and mucus production [103]. It was also established that human milk-derived exosomal miR-148a-3p prevents NEC by promoting Sirtuin 1 and inhibiting p53 and NF-κB expression [94]. Moreover, a study revealed that human milk-derived exosomes play a beneficial role in the prevention of NEC by reducing inflammation and injury of LPS-induced NEC of the intestinal epithelium, and also protect the integrity of the intestinal epithelial barrier, and also promote cell proliferation, as well as reduce the level of pro-inflammatory cytokines, and also increase the expression of the intestinal tight junction proteins [157]. A recent study indicated that human breast milk derived exosomes alleviate NEC associated intestinal injury and NEC ileal inflammation by reducing the NEC scores, restoring the number of damaged ileal crypts, and also inhibit the inflammatory responses of IECs [96], in addition, the human breast milk-derived exosomes prevent the development of NEC by reducing the expression of inflammatory cytokines such as TNFα and TLR4, as well as protecting the intestinal tract from epithelial inflammatory damage induced by LPS [158]. Furthermore, studies have established that porcine milk-derived exosomal miRNAs promote cell proliferation, inhibit the formation of tight junction proteins (TJs), and protect IECs from intestinal mucosal damage induced by DON [112]. Porcine milk-derived exosomal miRNAs such as miR-4334, miR-219, and miR-338 were reported to protect IEC damage induced by LPS by inhibiting apoptosis and inflammation via the p53 and TLR4/NF-κB pathways [113]. In other study, it was reported that bovine milk-derived exosomes enhance goblet cell activity and prevent the development of experimental NEC [159], furthermore, rat milk-derived exosomes exert several biological functions such as enhancing IEC activity, promoting cell proliferation, stimulating intestinal stem cell activity, and preventing the development of NEC in infants with breastfeeding intolerance [119].

### Colorectal cancer (CRC)

Colorectal cancer (CRC) is the third most common malignancy in the world, with an average of one person diagnosed with colorectal cancer every 1.5 min, resulting in nearly 900,000 deaths annually. With the process of urbanization and the aging population, the incidence and mortality cause by colorectal cancer is on the rise, therefore, developing ways to control and prevent the colorectal cancer disease is urgently needed. This is because, the symptoms of this disease only appear in advanced stages. Hence, several countries worldwide promote screening programs with the aim of increasing early detection rates of colorectal cancer in order to reduce morbidity and mortality [160–163]. Recent studies have reported that exosomes can be used as delivery vectors in vivo, to deliver valuable genetic cargo, containing biomarkers and load drugs for delivery to specific tissues, attracting an increasing interest because exosomes exert no adverse immune responses as well as prevent tumor formation [164, 165], hence, exosomes can be employed as potential biomarkers and target therapies for colorectal cancer [166]. Studies have shown that exosomal delivery of miR-128-3p is a novel strategy to enhance CRC chemical sensitivity through negative regulation of Bmi1 and MRP5 [90]. Exosomal delivered circRNAs promote glycolysis and chemotherapy resistance in CRC via the miR-122/PKM2 axis [167]. Exosomal circPACRGL promotes colorectal cancer proliferation and metastasis through the miR-142-3p/miR-506-3p-TGF-β1 axis [92]. Mesenchymal stem cells (MSCs)-derived exosomes contain tumor-inhibiting miRNAs (miR-3940-5p/miR-22-3p/miR-16-5p), which inhibits the proliferation, migration and invasion of CRC cells by regulating Ras-associated protein B2 (RAP2B)/phosphoinositide 3-kinase (PI3K)/AKT signaling pathway and integrin alpha 2/6 (ITGA2/6), thereby showing therapeutic potential in the UC and CRC [168]. Adipocyte derived exosomal microsomal triglyceride transfer protein (MTTP) inhibits ferroptosis and promotes chemotherapy resistance in CRC [2]. Tumor-derived exosomal miR-934 induces macrophage M2 polarization to enhance liver metastasis of CRC [169].

Due to its potential in preventing and treating CRC, milk is receiving increasing attention due to the abundance of exosomes it contains. Milk exosomes have been widely reported to exert direct antitumor effects on colorectal cancer. For instance, bovine milk-derived exosomes were reported to exhibit intrinsic antitumor activity by inhibiting the growth and activity of CRC cells, providing an effective alternative to oral administration for the treatment of CRC [170]. In addition, human milk-derived exosomes were shown to increase the expression level of miR-148a in the CRC cells but decrease the expression of its target gene DNA methyltransferase1 (DNMT1) to reduce the occurrence of CRC [171, 172]. It was also revealed in other studies that human milk-derived exosomes alter the miRNA expression profile of colon epithelial cells and also promote the proliferation of healthy colon epithelial cells without affecting the growth of CRC cells [173]. Furthermore, bovine milk-derived exosomes were also reported to attenuate the primary CRC by decreasing the number of CRC cell colonies as well as increase the cell death [117]. In goats, the milk-derived exosomes showed potential biological functions such as anti-inflammation, tumor retention, and increase production performance and high biosafety, and also act as ideal nanocarriers for the construction of CRC comprehensive diagnosis and treatment. The nanoprobes designed by goat milk-derived exosomes are used to trigger anti-tumor immune and inflammatory responses to enhance their potential in CRC therapy [174]. High levels of miR-27b in buffalo milk-derived exosomes exert their anti-CRC activity in vitro through the promotion of apoptosis of CRC cells, and increasing the accumulation of lysosome and mitochondrial reactive oxygen species (ROS), as well as aggravating the endoplasmic reticulum (ER) stress-mediated CRC cell death via protein kinase RNA-like ER kinase (PERK)/inositol-requiring enzyme 1 (IRE1)/X-box binding protein 1 (XBP1) and CHOP protein pathways [175].

### Intestinal ischemia and reperfusion injury (I/R)

Intestinal Ischemia/reperfusion (I/R) injury is a common clinical event caused by acute mesenteric ischemia, intestinal obstruction, intestinal transplantation and other pathophysiological factors, which cause micro vascular injury, mitochondrial oxidative damage and cell apoptosis [176, 177]. Due to the hidden onset and lack of effective treatment of I/R, the morbidity and mortality are high. Exploring strategies to reduce intestinal I/R injury is of great significance for improving organ recovery and patient survival [178, 179]. NLRX1/FUNDC1/NIPSNAP1-2 axis mediated mitophagy [180], live kinase B1 (LKB1)/AMPK mediated autophagy [181], and mtDNA-STING pathway [182] were reported as key mechanisms in the pathogenesis of intestinal I/R injury. Bone marrow mesenchymal stem cell-derived exosomes were found to ameliorate the intestinal I/R via the miR-144-3p-mediated oxidative stress and the phosphatase and tensin homolog (PTEN)/Akt/nuclear factor E2-related factor 2 (Nrf2) pathway [98], and also regulate the immune responses and attenuate neuronal apoptosis [183] and intestinal I/R injury-induced lung injury via the TLR4/NF-κB pathway [184]. During the intestinal I/R injury, gut-derived exosomes induce liver injury by promoting hepatic M1 macrophage polarization [185], mediate memory impairment by activating microglia [186]. In addition, the inhibition of the secretion of gut-derived exosome may be a therapeutic target for the prevention of hepatic impairment and memory impairment after the intestinal I/R. Human breast milk provides neonates with the protective and therapeutic for intestinal IR injury and NEC through deceasing the IL-1β-induced activation of NF-κB pathway [187]. Milk exosomes have the potential to cross physiological boundaries and cell membrane [188], however, exert no systemic toxic effects or anaphylaxis [189]. Human breast milk-derived exosomes alleviate intestinal damage in IR rats by reducing the intestinal hyperplasia and decrease the expression of an inflammatory cytokine TNFα [190].

**Fig. 4:**
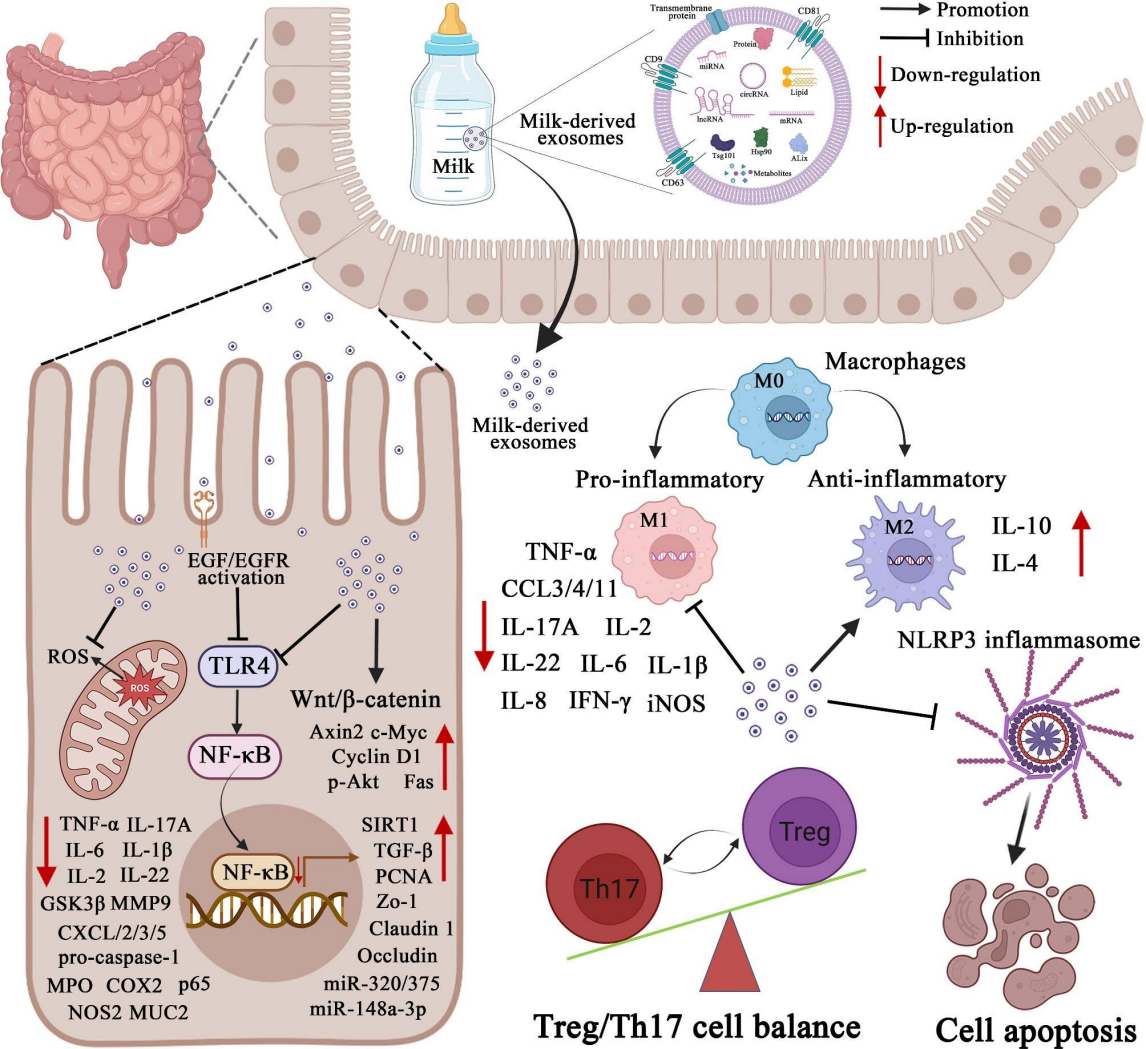
The regulatory mechanisms of milk-derived exosomes in the intestinal diseases

In general, it is increasingly clear that milk-derived exosomes are significantly involved in alleviating intestinal diseases, such as IBD, NEC, CRC, and intestinal IR injury, via regulating gut microbiota intestinal immune homeostasis, oxidative stress, inflammatory response, and proliferation and apoptosis (Fig. 4 and Table 1). The application of the exosomes based on their properties such as stability, transportability, and bioavailability, milk derived exosomes may be used as drug carriers for the transportation of drugs used for the treatment of targeted diseases.

**Table 1:** Milk-derived extracellular vesicles alleviate intestinal related diseases

Exosomal sources	Intestinal disease model	Mechanisms	Effect factors	Ref.
Cow milk	DSS-induced UC Mice	Alleviates colitis by regulating the intestinal immune homeostasis via inhibiting NLRP3 and TLR4-NF-κB signaling pathways, restoring the α-diversity of the gut microbiota effectively and Treg/Th17 cell balance.	IL-1β, TNF-α, IL-6, IL-2, and IL-22↓; IL-17 A, L-23R, MPO↓; TLR4, Myd88, iNOS, COX2, p-IκBα, p65↓; ASC, NLRP3, and pro-caspase-1↓; IL-10↑; *Akkermansia*↑	[136]
Cow milk	DSS-induced UC Mice	Modulates the inflammatory response through the interplay between the NFκB and A20, and restore a normal gut microbiota profile.	TNFAIP3, Zo-1↑; NFκB, COX2, and miR-125b↓; pro-inflammatory cytokines↓; anti-inflammatory cytokines↑	[137]
Cow and human milk	DSS-induced UC Mice	Attenuates the severity of colitis and reduce colon shortening, reduce the expression of pro-inflammatory cytokine.	TNF-α, IL-6, DNMT1/3↓; TGF-β, miR-320/375, and Let-7↑	[138]
Cow milk	DSS-induced UC Mice	Alleviates the severity of acute colitis, reduce the expression of pro-inflammatory cytokines, chemokine ligands and chemokine receptors.	CXCL2/3/5, CCL3/4/11↓; PTGS2, IL-1a, IL-1β, IL-33, IL-6 and IL-17 A↓	[139]
Cow milk	DSS-induced UC Mice	Attenuates colitis through optimizing gut microbiota abundance and by regulating the expression of the intestinal genes.	IL-6 and TNF-α↓; *Dubosiella*, *Bifidobacterium*, *UCG-007*, *Lachnoclostridium*, *Lachnospiraceae*↑; butyrate and acetate↑	[140]
Cow milk	DSS-induced UC Mice	Regulates the concentrations of lipids and amino acids in both fecal samples and colonic tissues, recover the metabolic abnormalities caused by inflammation.	Acetate, butyrate, L-arginine↑; C13:0, C15:1, C20:1, C20:2, C20:5, C22:6↓; L-valine, L-serine and L-glutamate↓	[141]
Goat milk	LPS-induced IPEC-J2 cells	Increases the antimicrobial peptides, defensins and toll like receptors, induce the preferential expression of the anti-inflammatory, improve intestinal homeostasis.	IL18, IL12p40, MMP9, NOS2↓; MUC2, EBI3, IL-6, IL-8↑	[142]
Human breast milk	LPS-induced NEC mice (intestinal epithelial cells, IEC-6 cells)	Protects against NEC and attenuate TLR4 signaling via EGF/EGFR activation, inhibit enterocyte apoptosis and restore enterocyte proliferation	TLR4, NF-κB, IL-6, IL-1β, GSK3β, iNOS↓; EGFR, PCNA↑	[150]
Human breast milk	hypoxia and gavage-induced NEC rat and human normal intestinal epithelial cell line (FHC)	Protects against NEC by promoting intestinal cell proliferation and migration	Ileum injury area↓; villous integrity, proliferation and migration↑	[152]
Human breast milk	H_2_O_2_-induced NEC (intestinal stem cells, ISCs)	Increases ISC viability, protect ISCs from oxidative stress injury via the Wnt/β-catenin signaling pathway	Axin2, c-Myc, and Cyclin D1↑	[153]
Human breast milk	LPS-induced NEC rat (intestinal epithelial cells, IEC-6 cells)	Decreases the incidence and severity of experimental NEC, increase IEC proliferation and decrease apoptosis, protect IEC from injury in vitro	Cell proliferation rate↑; Late apoptotic cells↓; intestinal damage↓; NEC incidence↓	[154]
Human breast milk	H_2_O_2_-induced NEC (intestinal epithelial cells, IECs)	Increases IEC viability, protects IECs from oxidative stress and cell toxicity induced by H_2_O_2_	cell viability↑	[155]
Human breast milk	hypoxia and gavage-induced NEC rat	reduce ischemic necrosis and epithelial damage, increase the number of BrdU-positive cells in the intestinal mucosa, decrease the severities of NEC	intestine length, number of BrdU-positive cells↑; NEC score↓	[95]
Human breast milk	LPS-induced NEC mice and human normal intestinal epithelial cell line (FHC)	Enhances epithelial cell proliferation and migration, and ameliorate the severity of LPS-induced NEC via ERK/MAPK pathway	gut damage and necrosis↓; NEC score↓; proliferation and migration↑; p-ERK↓	[156]
Human breast milk	Hypoxia, LPS-induced NEC mouse and intestinal epithelial cells (IEC)	Attenuates NEC damage by reducing the intestinal epithelial injury and inflammation, restoring the intestinal mucous production, and increasing goblet cells	IL-6↓; injury condition↓; injury score↓; MPO activity, MUC2↓; goblet cells↑	[103]
Human breast milk	LPS-induced NEC mice (Caco-2 and NCM460 cell lines)	Prevents NEC by reducing inflammation and injury in the intestinal epithelium as well as restores the intestinal tight-junction proteins	ZO-1, Claudin 1, and OCLN↑	[157]
Human breast milk	LPS-induced NEC mice (intestinal epithelial IEC6 cells)	Exerts significant protective effect on NEC mice, including inhibiting inflammation and cell apoptosis, and improving intercellular tight junctions	miR-148a-3p, SIRT1↑; p53, NF-κB↓	[94]
Human breast milk	asphyxia and cold stress-induced NEC mice and LPS-induced intestinal epithelial IEC-6 and IEC-18 Cell Lines	Attenuates the severity of experimental NEC and intestinal damage through reducing NEC score and ileal inflammation, restoring the number of damaged ileal crypts	Lgr5, MBP↑; IL-6, Iba1↓; NEC score↓; ileum crypts number↑; cell migration rate↑	[96]
Human breast milk	LPS-induced NEC C57BL/6 mice	Attenuates NEC-induced epithelial injury by reducing inflammation through inhibiting TNFα and TLR4 expression, and stimulating intestinal regeneration	TNF-α, TLR4, Ki67 and Lgr5↓;	[158]
Porcine milk	DON-induced NEC mice and porcine jejunum intestinal enterocytes IPEC-J2 cells	Protects the intestine against DON-induced damage by promoting cell proliferation and TJs and by inhibiting cell apoptosis	β-catenin, cyclin D1, p-Akt↑; ZO-1, OCLN, and CLDN1↑; p53, p21, Caspase 3, Caspase 9, Fas, and SERPINE1↓; miR-181a, miR-365-5p, miR-30c, and miR-769-3p↑	[112]
Porcine milk	LPS-induced NEC mice	Protects against the LPS-induced intestine epithelial cell injury by inhibiting cell apoptosis and inflammation through the p53 and TLR4/NF-κB pathway via the action of exosome miRNAs	IL-1β, IL-6, and TNF-α↓; p53, FAS, and Caspase-3↓; TLR4, Myd88, p-IκBα and p-NF-κB↓; miR-4334, miR-219, and miR-338↑	[113]
Bovine milk	LPS, hypoxia, and hyperosmolar formula feeding induced NEC mouse and human colonic LS174T cells	Prevents NEC-induced mouse intestinal injury by increasing goblet cell production and ER function	MUC2, TFF3, and GRP94↑; mucin production and goblets cell↑; MPO↓	[159]
Rat milk	Intestinal epithelial cells (IEC-18)	Prevents NEC by promoting IEC viability and proliferation, and stimulating intestinal stem cell activity	PCNA, Lgr5, and cell viability↑	[119]
Bovine milk	Human colon cancer (HCT116) cell lines	Inhibits colon cancer cell growth and survival, and anti-inflammatory activity, providing an effective alternative for oral delivery	cell growth and survival↓	[170]
Human breast milk	Colon epithelial cell line (CRL 1831)	Reduces risk of colon cancer by elevating the expression of miR-148a and decreasing DNA methyltransferase1	miRNA-148a↑; DNMT1↓	[171]
Human breast milk	Colonic epithelial cells (CCD 841) and colonic tumor cells (LS123)	Alter the miRNA expression profile of the colon epithelial cells and promote the proliferation of healthy colon epithelial cells without affecting the growth of the colon cancer cells	miR-148a↑; collagen-type I, PTEN, and DNMT↓	[173]
Bovine milk	Colorectal cancer cells (LIM1215, SW620)	Attenuates tumor burden through decreasing the number of colonies and increasing cell death in the colorectal cancer cells	Number of colonies↓; percentage of cell death↑; tumor volume↓	[117]
Goat milk	Mouse colon cancer cell line (MC38)	Enhances the antitumor effect of the photothermal therapy and reduce the inflammatory response after treatment	Ki67, TNF-α, IL-6, and IL-1β↓; tumor weights↓; CD3^+^CD4^+^ and CD3^+^CD8^+^↑;	[174]
Buffalo milk	Colorectal cancer cells HCT116, and HT-29	High expression of miR-27b induce higher cytotoxic effects, CRC cell apoptosis, ROS and lysosome accumulation via PERK/IRE1/XBP1 and CHOP protein modulation	ROS, PERK, IRE1, XBP1, ATF6, CHOP, Bax/Bcl-2, p-ERK/ERK, procaspase-12, p-p38/p38, and p-JNK/JNK↑; apoptosis, lysosome, ER-tracker↑	[175]
Human breast milk	Intestinal IR injury rats	Protects the intestine against damage from IR injury by decreasing the intestinal inflammation and enhancing epithelial proliferation	TNFα↓; Ki67↑; Intestinal IR injury score↓	[190]

↓: downregulation; ↑: upregulation.

## Milk exosome-based drug delivery systems for Disease therapy

Presently, drug delivery system is a novel area that many researchers are experimenting. This research area is rooted in the difficulty of treating some diseases with traditional therapeutic drugs and several drug delivery methods. Interestingly, exosomes can act as clinical drug carriers and they are also immune compatible. However, due to the lack of sources and methods for obtaining adequate exosomes, the therapeutic application of exosomes as drug carriers is limited. Milk-derived exosomes have several advantages such as higher yield, additional therapeutic benefits and oral delivery characteristic compared with other delivery vectors [191]. Milk-derived exosomes are highly biocompatible and remain intact after absorption in the gastrointestinal tract, indicating good stability. These properties make lacto-derived exosomes suitable drug carriers, but these lacto-derived exosomes already have substantial immunomodulatory functions on their own, and these vesicles can be used as therapeutic agents even when they are not loaded. However, milk exosomes show cross-species tolerance, no adverse immune and inflammatory responses, and further, milk exosomes are good drug deliverers, carrying cargo with tumor targeted therapy capabilities [192]. Multifunctional lacto-derived exosomes provide solutions to the challenges posed by the oral drug delivery, thus providing new insights into the development of oral drug delivery nanocarriers for natural equipment [193] [135]. Milk-derived exosome-loaded insulin (MDEI) elicited a more excellent and sustained hypoglycemic effect, the excellent oral delivery ability of MDEI attributed to versatile effects include high biocompatibility and bioavailability, active multi-targeting uptake, nutrient assimilation related ERK1/2 and p38 MAPK signal pathway activation, and intestinal mucus penetration, which is simple and cost-effective approach for the preparation of MDEI contributed to their large-scale production [193]. Studies have indicated that milk-derived exosomes serve as nanocarriers to deliver curcumin and resveratrol to breast tissues and enhance their anticancer activities [194], loaded with curcumin to improve the cell uptake and intestinal permeability of curcumin [195], and also act as agents for anticancer drug delivery [196], as well as have higher mucus penetration to improve the efficacy of the oral administration in the treatment of the intestinal bacterial infection. Natural flavonoid such as alpha-mangosteen was loaded into the milk exosomes and it was observed that it has eliminated approximately 99% of the bacteria in the macrophages [197], hence, milk-derived exosomes can be used as stable oral drug delivery carriers. Curcumin encapsulated in milk exosomes can resist human digestion and has enhanced in vitro intestinal permeability, and effectively penetrate the intestinal barrier [198]. Oral chemotherapy drug paclitaxel encapsulated in milk exosomes replaces conventional intravenous therapy to improve the efficacy and also reduce toxicity, thereby inhibiting the effect on tumor growth [199].

Recently, milk-derived exosomes have attracted attention as vehicles for delivering RNA therapeutics to cancers [200]. Milk-derived exosomes act as a novel system for the delivery of miR-31-5p, and also successfully encapsulated miR-31-5p mimics into milk exosomes through electroporation dramatically to improve the endothelial cell functions in vitro and promote the angiogenesis and also enhance the diabetic wound healing in vivo [201]. Bovine milk is a cost-effective source of potential exosomes which can be used as nanocarriers of functional drug delivery vehicle for miRNA-based therapy, exosome-transported miR-148a-3p can be delivered and taken up by cells *in-vitro*, and exert a biological effect through the modulation of gene expression [202]. Milk-derived exosomes can be used as a natural nanoparticles for novel small interfering RNA (siRNA) delivery system, and can enhance mucus penetrability and penetrated multiple biological barriers for oral drug delivery of siRNA [203, 204], and delivered endogenous RNA payloads into the recipient cells, and loaded siRNA against specific genes such as KRAS which represents a viable natural nano-carrier for the delivery of siRNA for the therapeutic application against cancer [205]. Milk-derived exosomes carrying siRNA-KEAP1 promote diabetic wound healing by alleviating oxidative stress [206].

Milk-derived exosomes have high concentration and diversity of cargos, which cross the blood-brain barrier and are absorbed and accumulated in tissues following oral administrations to deliver drugs to the diseased tissues [207]. Milk-derived exosome as an oral drug delivery system with a great application potential improve drug safety, bioavailability, and effectiveness in the delivery of the oral preparations [208]. Milk-derived exosomes encapsulated doxorubicin can penetrate the tumor and delivery to triple-negative breast cancer cells would be effective in reducing triple-negative breast cancer cells’ survival [209]. Hyaluronic acid-coated bovine milk exosomes for tumor-specific delivery of miR-204 showed an excellent biocompatibility and exert no significant systemic toxicity, but significantly increased antitumor efficacy both in vitro and in vivo. Both hyaluronic acid and bovine milk-derived exosomes are low-cost and highly accessible biogenic materials with broad biomedical applications. The hyaluronic acid-decorated bovine milk-derived exosomes are proven as practical drug delivery system of RNA drugs for targeted cancer therapy [210]. An in vitro experiment indicated that doxorubicin-loaded milk-derived exosomes with hyaluronic acid triggers tumor cell death, and therefore, demonstrates its potential use for tumor cell-specific drug delivery and feasible for targeted cancer therapy [211]. A study by Zhang et al. proved that milk-derived exosomes-based drug delivery system showed controlled drug-release and biocompatibility, hence, they are effective in treating oral squamous cell carcinomas [212]. In addition, milk-derived exosomes encapsulation of hydrophilic biomacromolecule drugs could significantly improve the transepithelial transport and bioavailability of the oral drugs [213]. Milk-derived exosomes encapsulated with forsythiaside A combats liver fibrosis via regulating NLRP3-mediated pyroptosis [214]. This shows that milk-derived exosomes exert several advantages, such as no adverse immune and inflammatory responses, and have great application potential in the treatment of targeted diseases by clinical drug delivery systems.

## Conclusions and future perspectives

Exosome is widely involved in the progression of various diseases, and plays an important role in disease diagnosis and also act as a drug carrier. In this comprehensive review, we summarized the biogenesis, secretion and structure, current methods for the extraction, and identification methods and markers of exosomes, and further highlighted the biological roles of the milk-derived exosomes in preventing and treatment of intestinal diseases, such as inflammatory bowel disease, necrotizing enterocolitis, colorectal cancer, and intestinal ischemia and reperfusion injury via the regulation of intestinal immune homeostasis, restoring gut microbiota composition and promote the intestinal mucous production, by alleviating oxidative stress, cell apoptosis and inflammation, as well as reducing the ROS and lysosome accumulation.

Milk-derived exosomes have been confirmed to exert no adverse immune and inflammatory responses, nontoxicity, high biocompatibility and bioavailability and has the potential of mass production for clinical therapy for various targeted diseases. However, further studies are required to establish and promote the standardization production platform of exosomes in milk to improve the utilization and obtain higher concentration and purity and more complete exosomes obtained from milk. In addition, several clinical in vivo studies should be carried out to explore the pharmacological effects and the pharmacokinetics of the milk-derived exosome-based drug delivery carriers for the therapy of targeted diseases, thereby to establish milk-derived exosomes as a mature drug delivery system and promote its widely use in the treatment of various diseases. Taken together, the use of milk-derived exosomes is useful in preventing and treating diseases in both humans and animals. Studies on the dietary supplementation of milk-derived exosomes could alleviate piglet diarrhea post-weaning and proliferative enteropathy in pigs require further exploration.

## Data Availability

Not applicable.

## Abbreviations

AMPK: adenosine monophosphate-activated protein kinase
ASC: apoptosis-associated speck-like protein
ATF6: activating transcription factor 6
Axin2: axis inhibition protein 2
C13:0: tridecanoic acid
C15:1: methyl cis-10-pentadecenoate
C20:1: cis-11-eicosenoic acid
C20:2: eicosadienoic acid
C20:5: eicosapentaenoic acid
C22:6: docosahexaenoic acid
CCL3/4/11: CC chemokine ligand 3/4/11
CHOP: CCAAT-enhancer-binding homologous protein
CLDN1: claudins 1
COX2: cyclooxygenase-2
CXCL2/3/5: C-X-C motif chemokine ligand
DNMT1: DNA methyltransferase1
EBI3: epstein-barr virus-induced gene 3
EGF: epidermal growth factor
ER: endoplasmic reticulum
GRP94: glucose-regulated protein 94
GSK3β: glycogen synthase kinase-3beta
Iba1: ionized calcium binding adaptor molecule 1
IFN-γ: interferon-gamma
IL-6: interleukin 6
iNOS: inducible nitric oxide synthase
IRE1: inositol-requiring enzyme 1
JNK: c-Jun N-terminal kinase
Lgr5: Leucine-rich repeat-containing G-protein coupled receptor 5
MDEs: milk-derived exosomes
MBP: myelin basic protein
MMP9: matrix metalloproteinase 9
MPO: myeloperoxidase
MUC2: mucin 2
NFκB: nuclear factor kappaB
NLRP3: nod-like receptor family pyrin domain containing 3
NOS2: nitric oxide synthase
OCLN: occludin
PCNA: proliferating cell nuclear antigen
PERK: protein kinase RNA-like ER kinase
PTEN: phosphatase and tensin homolog
PTGS2: prostaglandin-endoperoxide synthase 2 (also known as COX-2)
ROS: reactive oxygen species
SERPINE1: serine protease inhibitor clade E member 1
SIRT1: sirtuin 1
TFF3: trefoil factor 3
TGF-β: transforming growth factor β
TLR4: Toll-like receptor 4
TNFAIP3: TNF-alpha-induced protein 3
TNF-α: tumour necrosis factor alpha
XBP1: X-box binding protein 1
Zo-1: zona occludens 1
